# PRN: progressive reasoning network and its image completion applications

**DOI:** 10.1038/s41598-024-72368-1

**Published:** 2024-10-09

**Authors:** Yongqin Zhang, Xiaoyu Wang, Panpan Zhu, Xuan Lu, Jinsheng Xiao, Wei Zhou, Zhan Li, Xianlin Peng

**Affiliations:** 1 School of Archaeology and Cultural Heritage, Zhengzhou University, Zhengzhou, 450001 China; 2 School of Information Science and Technology, Northwest University, Xi’an, 710127 China; 3 Information and Data Department, Shaanxi History Museum, Xi’an, 710061 China; 4 Electronic Information School, Wuhan University, Wuhan, 430072 China; 5 Art School, Northwest University, Xi’an, 710127 China

**Keywords:** Image completion, Image inpainting, Deep learning, Ancient murals, Pigment shedding, Applied mathematics, Computational science, Computer science, Information technology, Software, Statistics, Mathematics and computing, Optics and photonics, Engineering, Electrical and electronic engineering

## Abstract

Ancient murals embody profound historical, cultural, scientific, and artistic values, yet many are afflicted with challenges such as pigment shedding or missing parts. While deep learning-based completion techniques have yielded remarkable results in restoring natural images, their application to damaged murals has been unsatisfactory due to data shifts and limited modeling efficacy. This paper proposes a novel progressive reasoning network designed specifically for mural image completion, inspired by the mural painting process. The proposed network comprises three key modules: a luminance reasoning module, a sketch reasoning module, and a color fusion module. The first two modules are based on the double-codec framework, designed to infer missing areas’ luminance and sketch information. The final module then utilizes a paired-associate learning approach to reconstruct the color image. This network utilizes two parallel, complementary pathways to estimate the luminance and sketch maps of a damaged mural. Subsequently, these two maps are combined to synthesize a complete color image. Experimental results indicate that the proposed network excels in restoring clearer structures and more vivid colors, surpassing current state-of-the-art methods in both quantitative and qualitative assessments for repairing damaged images. Our code and results will be publicly accessible at https://github.com/albestobe/PRN.

## Introduction

Ancient murals, as invaluable cultural relics, provide crucial supplementary insights into historical documents. However, due to natural and man-made destruction, these artworks are often subjected to various forms of degradation, including pigment shedding, cracking, mildew, and mud pollution. Such damage significantly impedes people’s comprehension and appreciation of these murals, diminishing their sense of understanding, enjoyment, and contentment. Conventionally, cultural relic restorers engage in the laborious and inefficient task of manually repairing mural paintings. This traditional approach falls short of meeting the demands of large-scale mural restoration and permanent preservation. In contrast, digital completion presents a potentially viable solution for virtual restoration. This technique enables the filling of missing areas in damaged mural images without physically altering the murals themselves, offering a promising alternative for preserving and appreciating these ancient artworks.

Over the past decade, deep learning-based completion methods^1–3^ have attracted significant attention due to their remarkable results in restoring natural images. These methods, primarily based on the codec framework, utilize an encoder to extract compact underlying features from damaged images and a decoder to reconstruct the entire image. Nevertheless, when applied to mural images, they often yield unsatisfactory outcomes due to data shifts and limited modeling efficacy. In their previous study, Zhang et al.^4^ proposed a content-constrained convolutional network for completing mural images by integrating dual-domain partial convolution and a space-varying activation function. However, they neglected the crucial aspect of the mural painting process, which typically commences with sketching and then proceeds to color. In this paper, we propose a novel progressive reasoning network (PRN) for restoring images of ancient murals by considering the mural painting process. The proposed network integrates two recursive double-codec modules and a paired-associate learning module. This network first estimates luminance and sketch maps from a damaged mural image and then merges them to restore the complete color image. We implemented and evaluated our PRN, comparing it with baseline methods on benchmark datasets. The experiments reveal that our PRN achieves superior repair results, outperforming baseline methods both qualitatively and quantitatively. The key contributions of this paper are threefold: (1) A novel progressive reasoning network is designed for mural image completion; (2) Two complementary double-codec modules are constructed to infer luminance and sketch maps, respectively; and (3) A paired-associate learning module is developed to synthesize the complete color image.

## Related work

### Early methods

Image completion, which dates back to the 2000s, aims to restore damaged or missing parts of an image to construct a visually complete image. Early image completion methods are broadly classified into two categories: diffusion-based and example-based approaches. Diffusion-based approaches^5,6^ rely on neighboring pixels surrounding the missing areas to propagate information inward to fill the holes. However, they are usually constrained to small or narrowly defined areas because of their inherent gradual pixel diffusion nature. In contrast, example-based methods^7,8^ search for similar image blocks either within the damaged image itself or in an external database to repair the damaged areas. While these approaches offer more flexibility, they rely heavily on the availability of matching image blocks, which can be challenging for complex structures and patterns. As a result, they may not be suitable for all types of damage, especially those with intricate details.

### Contemporary methods

Unlike early methods, contemporary image completion methods use neural networks to capture semantic information, facilitating the restoration of damaged or missing image areas. Recent advancements in computer hardware and computing power have spurred the development of numerous deep learning-based completion methods. Pathak et al.^9^ introduced a context encoder network that incorporates both an encoder-decoder framework and adversarial learning for image completion. However, this approach only enforces constraints on filled areas through adversarial loss, neglecting global consistency, which can lead to distorted boundaries. To enhance the overall realism of repaired images, Iizuka et al.^10^ integrated a global discriminator into the context encoder network, albeit with limitations in restoring intricate textures and details. To suppress blur and visual artifacts in repaired areas, Yang et al.^11^ presented a multi-scale neural patch synthesis method that optimizes both image content and texture constraints. Song et al.^12^ presented a two-step context-based neural network that separates the image completion task into inference and translation, ensuring visually coherent completion.

Conventionally, convolutional neural networks treat both damaged and intact areas identically, which can result in blurring artifacts and color aberrations in repaired images. To address this issue, Liu et al.^13^ proposed a partial convolutional network (PCN), which utilizes an automatic mask updating mechanism to constrain convolution operations to valid pixels. Zhang et al.^14^ decomposed image completion into multiple sub-tasks connected through a long short-term memory (LSTM) framework^15^, enabling step-by-step repairs from the boundaries of missing areas towards the interior. Shen et al.^16^ presented a densely connected generative network designed for single-shot semantic image completion. Hong et al.^17^ integrated feature fusion blocks into the decoding path of U-Net, ensuring smoother transitions at the boundaries of filled areas. To address holes overlapping or touching foreground objects, Xiong et al.^18^ proposed a foreground-aware image completion technique that explicitly disentangles structure inference and content completion. Recognizing that missing areas may encompass multiple semantic categories, Liao et al.^19^ introduced a joint optimization framework for image segmentation and completion, leveraging coherent priors between semantics and textures. Shin et al.^20^ introduced a lightweight and efficient semantic completion network that utilizes parallel extended-decoder paths to improve completion performance and reduce hardware costs. To address large holes in complex scenes, Zhou et al.^21^ introduced a reference-guided image completion method that integrates multi-homography, deep warping, and color harmonization. Kang et al.^22^ developed a completion neural network capable of generating 3D images from sparsely sampled 2D images. To minimize structural distortions and texture blurring artifacts in repaired images, Zeng et al.^23^ proposed an aggregated contextual transformation method specifically designed for high-resolution image completion. To synthesize visually coherent content for missing regions, Shamsolmoali et al.^24^ presented a context-adaptive transformer for image completion. Shao et al.^25^ proposed a damage attention graph module to estimate the damage degree of mural images. A series of loss functions are used to adaptively select repair strategies based on the diversity of damage. To balance long-range modeling capabilities with computational efficiency, Huang et al.^26^ introduced a sparse self-attention transformer tailored for image completion tasks. Seeking to eliminate the need for domain-specific training while maintaining fast inference speeds, Corneanu et al.^27^ presented a diffusion model that incorporates forward-backward fusion in latent space for image completion. Xu et al.^28^ proposes a united image completion method by integrating the UNet framework and the diffusion model, which first detects cracks in murals and then repairs them. Wei et al.^29^ presented a two-stage restoration model for mural images under low light and defective conditions. Although these methods have achieved impressive results on natural images, they often yield unsatisfactory outcomes when applied to mural images due to data shifts and model inefficiencies. Mural images are characterized by abundant lines and smooth colors, exhibiting distinct patterns different from those found in natural images. Furthermore, the availability of mural images is limited in practice. As a result, these methods tend to produce unnatural repair appearances and severe artifacts, especially in the cases of large missing areas. In this study, we will present an efficient progressive reasoning network for completing mural images. This network infers image luminance, sketch, and color to facilitate comprehensive image restoration.

## Method

In this section, we introduce the PRN model specifically designed for completing mural images. This model first infers a pair of luminance and sketch maps and then merges them to restore the complete color image. We will elaborate on the network architecture, the loss function, and other relevant details.

### Network architecture

**Fig. 1:**
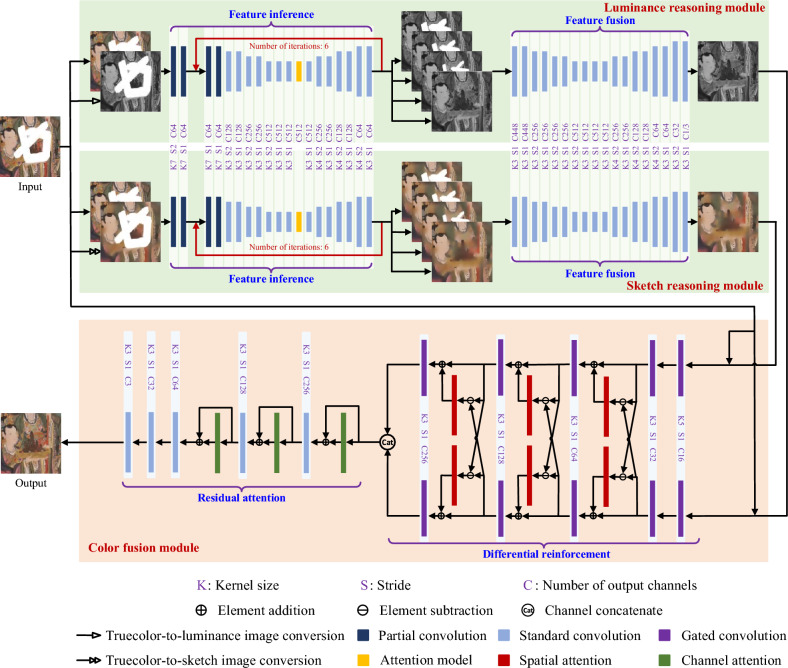
Network architecture of our PRN.

Figure 1 depicts the architecture of our PRN, which consists of two stages comprising three modules: a luminance reasoning module, a sketch reasoning module, and a color fusion module. In the first stage, both the luminance and sketch reasoning modules are constructed using the cyclic double-codec framework. The luminance reasoning module receives a damaged image along with its corresponding luminance image as inputs, ultimately generating a repaired luminance map as the output. Concurrently, the sketch reasoning module processes the damaged image and its corresponding sketch image, resulting in a repaired sketch map as the output. These luminance and sketch images are derived from the true-color damaged image by converting it to a luminance image and applying bilateral filtering^30^ to the original image, respectively. The luminance image represents light intensity, revealing the reflectance properties of mural surfaces, while the sketch image emphasizes image edges and partial color information. In the second stage, the color fusion module, grounded in the paired-associate learning framework, integrates the original damaged image with its luminance and sketch maps to produce the repaired image as the final output. In the upcoming subsections, we will delve deeper into the specifics of these three modules.

#### Luminance and sketch reasoning modules

The first stage of the PRN includes luminance and sketch reasoning modules. Both modules share an identical cyclic double-codec framework. However, they serve distinct purposes: one infers luminance information for missing areas, while the other infers sketch details. For the luminance reasoning module, the label is the ground-truth luminance map, while the inputs consist of the damaged image and its corresponding luminance image. Conversely, the sketch reasoning module uses the ground-truth sketch map as the label, with the damaged image and its corresponding sketch image as the inputs. Inferring luminance and sketch maps separately proves simpler than attempting to directly deduce all information from the complete true-color image. Each module estimates image data from the outside to the inside of the missing areas. This process involves feature inference and feature fusion blocks, corresponding to the former and latter codecs, respectively. More precisely, feature inference operates iteratively. It first employs partial convolution to identify a ring-shaped region at the boundaries of each missing area and then fills this ring during each iteration until all missing areas are completed. Meanwhile, feature fusion combines all iteration outputs from the previous codec and then feeds them into the subsequent codec to generate either the luminance or sketch map.

##### Feature inference

The feature inference block adopts a cyclic U-shaped codec structure that operates iteratively until all missing areas are filled. The first two layers of this former codec consist of partial convolutional layers, which aim to fill the ring-shaped regions on the boundaries of the missing areas during each iteration. These partial convolutional layers not only operate on valid regions of feature maps but also dynamically update the corresponding binary mask. Let ${{\textbf{W}}_k}$ and *b* represent the weights for the *k*-th channel of the convolution filter and its bias, respectively. Within the current sliding convolution window, both ${{\textbf{X}}_{i,j}}$ and ${{\textbf{M}}_{i,j}}$ represent the input feature (or pixel values) and the corresponding binary mask, respectively. The output feature value generated by the partial convolutional layer at location (*i*, *j*, *k*) can be formulated as:

1 $$ x^{\prime}_{{i,j,k}}  = \left\{ {\begin{array}{*{20}l}    {\frac{{{\mathbf{W}}_{k}^{T} ({\mathbf{X}}_{{i,j}}  \odot {\mathbf{M}}_{{i,j}} )S\left( {\mathbf{1}} \right)}}{{S\left( {{\mathbf{M}}_{{i,j}} } \right)}} + b,} & {S\left( {{\mathbf{M}}_{{i,j}} } \right) > 0}  \\    {0,} & {S\left( {{\mathbf{M}}_{{i,j}} } \right) = 0}  \\   \end{array} } \right., $$

where *T* denotes the transpose operation, $\odot$ represents the element-wise multiplication, $\textbf{1}$ is the all-ones matrix of the same size as ${{\textbf{M}}_{i,j}}$, and $S\left( {\textbf{M}}_{i,j} \right)$ calculates the sum of all elements in the hole mask $\textbf{M}_{i,j}$. Following each partial convolution, the mask ${{\textbf{M}}_{i,j}}$ is updated as follows:

2 $$ m^{\prime}_{{i,j}}  = \left\{ {\begin{array}{*{20}c}    {1,} & {S\left( {{\mathbf{M}}_{{i,j}} } \right) > 0}  \\    {0,} & {S\left( {{\mathbf{M}}_{{i,j}} } \right) = 0}  \\   \end{array} } \right., $$

where ${m'_{i,j}}$ represents the updated pixel value at location (*i*, *j*) in the mask.

Within the bottleneck of the U-shaped codec, we introduce an attention layer to model the visual saliency of images. This attention layer leverages the complementarity of similar features to fill in missing areas with realistic textures. Specifically, we first measure the similarity between any two distinct vectors ${\textbf{x}_{i,j}}$ and ${\textbf{x}_{i', j'}}$ in feature maps at the $\tau$-th iteration:

3 $$\begin{aligned} \hat{s}_{i, j, i', j'}^\tau = \left\langle {\frac{{{{\textbf{x}}_{i,j}}}}{{\left\| {{{\textbf{x}}_{i,j}}} \right\| }},\frac{{{{\textbf{x}}_{i', j'}}}}{{\left\| {{{\textbf{x}}_{i', j'}}} \right\| }}} \right\rangle , \end{aligned}$$

where $\left\| \cdot \right\|$ denotes the Euclidean norm, and $\langle \cdot \rangle$ indicates the cosine similarity measure. Then we average the similarity values in a square neighborhood centered at location (*i*, *j*) in this formula:

4 $$\begin{aligned} {\bar{s}_{i,j,i',j'}^\tau = \frac{1}{{{{(2r + 1)}^2}}}\sum \limits _{p,q \in \left\{ { - r, \cdots ,r} \right\} } {\hat{s}_{i + p,j + q,i',j'}^\tau },} \end{aligned}$$

where $2r+1$ is the side length of the square neighborhood. Next, we normalize these similarity values to compute the attention score:

5 $$\begin{aligned} \hat{a}_{i,j,i',j'}^\tau = \frac{{\bar{s}_{i,j,i',j'}^\tau }}{{\sum \limits _{i' \in \left\{ {1, \cdots ,W} \right\} ,j' \in \left\{ {1, \cdots ,H} \right\} } {\bar{s}_{i,j,i',j'}^\tau } }}, \end{aligned}$$

where *W* and *H* are the width and height of the feature maps, respectively. Since the feature maps pass through the attention layer at each iteration, for a pixel located in valid regions (i.e., with a mask value of 1) at the $\left( {\tau -1}\right)$-th iteration, we calculate the weighted score by considering two consecutive iterations:

6 $$\begin{aligned} a_{i,j,i',j'}^\tau = \lambda \hat{a}_{i,j,i',j'}^\tau + \left( {1 - \lambda } \right) \hat{a}_{i,j,i',j'}^{\tau - 1}, \end{aligned}$$

where $\lambda \in \left[ {0,1}\right]$ is a weighting parameter. For invalid areas, we use the attention score solely from the current iteration:

7 $$\begin{aligned} a_{i,j,i',j'}^\tau = \hat{a}_{i,j,i',j'}^\tau . \end{aligned}$$

Finally, we use the weighted scores to compute the feature value at location $\left( {i,j} \right)$ as follows:

8 $$\begin{aligned} {{\hat{\mathbf{x}}}}_{i,j}^\tau = \sum \limits _{i' \in \left\{ {1, \cdots , W} \right\} ,j' \in \left\{ {1, \cdots ,H} \right\} } {a_{i,j,i',j'}^\tau \mathbf{{x}}_{i',j'}^\tau }. \end{aligned}$$

We collect these feature values to construct attention-corrected feature maps ${{{\hat{\mathbf{X}}}}^\tau }$, and then feed both ${{{\hat{\mathbf{X}}}}^\tau }$ and ${\mathbf{{X}}^\tau }$ into the subsequent convolutional layer in the decoder of the codec.

Throughout the iterative process of feature inference, the mask is continuously updated until all its values become 1, indicating that the feature maps have been fully generated without any missing areas.

##### Feature fusion

 The feature fusion block is another U-shaped codec designed to aggregate all outputs from feature inference for accurate luminance or sketch map estimations. To mitigate the influence of invalid values, we utilize the element-wise product of the output feature maps and their corresponding masks derived from feature inference as the inputs for feature fusion. The output $\mathbf{{Y}}$ of feature fusion can be formulated as:

9 $$\begin{aligned} \mathbf{{Y}}\!=\!{\varphi _{{F_2}}}\left( {{\mathbf{{X}}^1}\!\odot \!{\mathbf{{M}}_1},\!\cdots \!,{\mathbf{{X}}^\tau }\!\odot \!{\mathbf{{M}}_\tau },\!\cdots \!,{\mathbf{{X}}^N}\!\odot \!{\mathbf{{M}}_N}} \right) , \end{aligned}$$

where ${\mathbf{{X}}^\tau }$ and ${\mathbf{{M}}_\tau }$ denote the output feature maps and their corresponding masks generated during feature inference at the $\tau$-th iteration, *N* designates the preset number of iterations, and ${\varphi _{{F_2}}}$ is the mapping function of feature fusion.

#### Color fusion

With the estimated luminance and sketch maps available, the color fusion module serves as the second stage of the PRN to synthesize the complete true-color image. This module is based on a paired-associate learning framework, incorporating blocks of both differential reinforcement (DR) and residual attention (RA) blocks. The DR block utilizes two interactive complementary streams to extract deep features from the estimated luminance and sketch maps, respectively. Meanwhile, the RA block merges the combined features to generate a realistically repaired image.

Given the presence of invalid areas with zero-value pixels and human visual saliency in damaged images, the DR block is designed using gated convolution^31^ and spatial attention^32^. It consists of five pairs of gated convolutional layers, three butterfly-shaped sections, and a concatenation layer. Unlike standard convolution treating both valid and invalid pixels equally, gated convolution dynamically selects features across all pixels at spatial channel locations. Let $\textbf{Y}_I$ and $\textbf{Y}_O$ represent the input and output of gated convolution, respectively. The output can be expressed as follows:

10 $$\begin{aligned} {{\mathbf{{Y}}_O} = \phi \left( {{\mathbf{{B}}_g} * {\mathbf{{Y}}_I}} \right) \odot \sigma \left( {{\mathbf{{B}}_f} * {\mathbf{{Y}}_I}} \right) }, \end{aligned}$$

where $*$ denotes the convolution operator, $\odot$ represents the element-wise multiplication operator, $\mathbf{{B}}_g$ and $\mathbf{{B}}_f$ are two distinct convolving kernels, $\phi \left( \cdot \right)$ is the activation function, and $\sigma \left( \cdot \right)$ is the sigmoid function.

**Fig. 2:**
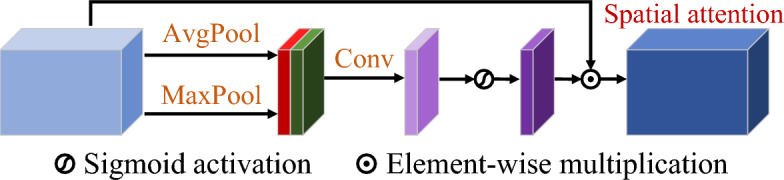
Internal structure of spatial attention layer.

Each stream in the DR block utilizes two cascaded gated convolutional layers to extract features from a pair of damaged images and the estimated luminance (or sketch) map. Subsequently, three cascaded pairs of butterfly-shaped sections and gated convolutional layers facilitate interactive communication and coordination of feature representation. For each butterfly-shaped section, let ${\mathbf{{F}}_{\mathrm{{Lumi}}}}$ and ${\mathbf{{F}}_{\mathrm{{Sketch}}}}$ represent its input luminance and sketch features, respectively. Then its outputs are given by:

11 $$\begin{aligned} {{{\mathbf{{F'}}}_{\mathrm{{Lumi}}}}\mathrm{{ = }}{\varphi _{\mathrm{{SP + }}}}\left( {{\mathbf{{F}}_{\mathrm{{Sketch}}}} - {\mathbf{{F}}_{\mathrm{{Lumi}}}}} \right) + {\mathbf{{F}}_{\mathrm{{Lumi}}}},} \end{aligned}$$

and

12 $$\begin{aligned} {{{\mathbf{{F'}}}_{\mathrm{{Sketch}}}}\mathrm{{ = }}{\varphi _{\mathrm{{SP - }}}}\left( {{\mathbf{{F}}_{\mathrm{{Lumi}}}} - {\mathbf{{F}}_{\mathrm{{Sketch}}}}} \right) + {\mathbf{{F}}_{\mathrm{{Sketch}}}},} \end{aligned}$$

where ${\varphi _{\mathrm{{SP + }}}}\left( \cdot \right)$ and ${\varphi _{\mathrm{{SP - }}}}\left( \cdot \right)$ denote two paired spatial attention layers that recalibrate features in the spatial domain to model visual saliency of images. Figure 2 illustrates the internal structure of the spatial attention layer.

Given an input feature map ${\mathbf{{F}}_\mathrm{{Diff}}} \in {{\mathbb {R}}^{H \times W \times C}}$, the output ${\mathbf{{F}}_\mathrm{{SA}}}$ of the spatial attention layer is

13 $$\begin{aligned} {\mathbf{{F}}_\mathrm{{SA}}} = {\mathbf{{A}}_{\mathrm{{SP}}}} \odot {\mathbf{{F}}_{\mathrm{{Diff}}}} \end{aligned}$$

with the spatial attention score ${\mathbf{{A}}_{\mathrm{{SP}}}}$ defined as

14 $$\begin{aligned} {\mathbf{{A}}_{{\text {SP}}}} = \rho \left( {{g_{{\text {Conv}}}}\left( {\left[ {{g_{{\text {Avg}}}}\left( {{\mathbf{{F}}_{{\text {Diff}}}}} \right) ;{g_{Max}}\left( {{\mathbf{{F}}_{{\text {Diff}}}}} \right) } \right] } \right) } \right) , \end{aligned}$$

where ${g_{{\text {Avg}}}}\left( \cdot \right)$ and ${g_{{\text {Max}}}}\left( \cdot \right)$ represent average pooling and maximum pooling along the channel direction, respectively. ${g_{{\text {Conv}}}}\left( \cdot \right)$ is a convolution operation with a kernel size of $\left[ {7,7} \right]$. $\rho \left( \cdot \right)$ denotes the sigmoid activation function.

**Fig. 3:**
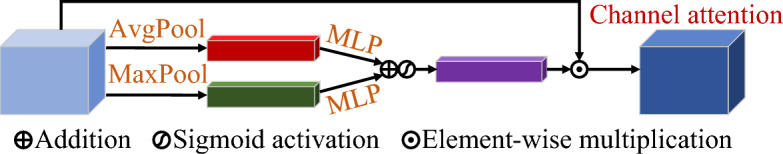
Internal structure of channel attention layer.

After passing through two interactive streams in the DR block, the concatenated deep feature maps are fed into the subsequent RA block. To synthesize a realistic complete image, the RA block incorporates residual learning^33^ and channel attention^34^. It consists of five standard convolutional layers, three-channel attention layers, and three residual skip connections. Channel attention complements spatial attention in modeling visual saliency by adaptively selecting and adjusting features in the channel domain. Figure 3 depicts the internal structure of the channel attention layer.

Given an input feature map ${\mathbf{{F}}_\mathrm{{In}}} \in {{\mathbb {R}}^{H \times W \times C}}$, the output ${\mathbf{{F}}_\mathrm{{CA}}}$ of the channel attention layer is expressed as

15 $$\begin{aligned} {\mathbf{{F}}_{{\text {CA}}}} = {\mathbf{{A}}_{{\text {CH}}}} \odot {\mathbf{{F}}_{{\text {In}}}} \end{aligned}$$

with the channel attention map ${\mathbf{{A}}_{{\text {CH}}}}$ defined as

16 $$\begin{aligned} {{\textbf{A}}_{{\text {CH}}}} = \rho \left( {{g_{{\text {MLP}}}}\left( {{g_{{\text {Avg}}}}\left( {{{\textbf{F}}_{{\text {In}}}}} \right) } \right) + {g_{{\text {MLP}}}}\left( {{g_{{\text {Max}}}}\left( {{{\textbf{F}}_{{\text {In}}}}} \right) } \right) } \right) , \end{aligned}$$

where ${g_{{\text {Avg}}}}\left( \cdot \right)$ and ${g_{{\text {Max}}}}\left( \cdot \right)$ represent average pooling and maximum pooling in the spatial domain, respectively. ${g_{{\text {MLP}}}}\left( \cdot \right)$ denotes a multi-layer perceptron composed of two fully connected layers. $\rho \left( \cdot \right)$ is the sigmoid activation function.

### Loss functions

We incorporate both perceptual and style losses into the respective loss functions of the luminance reasoning, sketch reasoning, and color fusion modules. These losses effectively measure the deep feature discrepancies between the predicted and ground-truth maps. Typically, the pre-trained VGG-16 model^35^ is used to extract relevant deep features for constructing loss functions. Let ${\Phi _{{P_m}}}$ represent the output feature maps of size ${H_m} \times {W_m} \times {C_m}$ from the *m*-th pooling layer of VGG-16. The perceptual loss is then formulated as follows:

17 $$\begin{aligned} {L_{\mathrm{{Percept}}}} = \sum \limits _{n = 1}^N {\frac{1}{{{H_m}{W_m}{C_m}}}{{\left\| {\Phi _{{P_m}}^{\mathrm{{GT}}} - \Phi _{{P_m}}^{\mathrm{{Pred}}}} \right\| }_1}}, \end{aligned}$$

where ${\left\| \cdot \right\| _1}$ denotes the ${\ell _1}$ norm, and $\Phi _{{P_m}}^{\mathrm{{Pred}}}$ and $\Phi _{{P_m}}^{\mathrm{{GT}}}$ represent the deep features extracted from the predicted and ground-truth maps, respectively. Similarly, the style loss is defined as:

18 $$\begin{aligned} \begin{aligned} {L_{\mathrm{{Style}}}}&= \sum \limits _{n = 1}^N {\frac{1}{{{C_m} \times {C_m}}}}\times {\frac{1}{{H_m}{W_m}{C_m}}} \\&\quad \times {\left\| {\Phi _{{P_m}}^{\mathrm{{GT}}}}{{\left( {\Phi _{{P_m}}^{\mathrm{{GT}}}} \right) }^T}-{\Phi _{{P_m}}^{\mathrm{{Pred}}}}{{\left( {\Phi _{{P_m}}^{\mathrm{{Pred}}}} \right) }^T} \right\| }_1. \\ \end{aligned} \end{aligned}$$

Furthermore, we incorporate the ${\ell _1}$ loss on valid areas (abbreviated as ${L_{\mathrm{{Valid}}}}$) and damaged areas (abbreviated as ${L_{\mathrm{{Hole}}}}$). The total loss for the luminance reasoning module is:

19 $$\begin{aligned} {L_{{\text {Lumi}}}} \!=\! {\lambda _1}L_{{\text {Percept}}}^{{\text {Lumi}}} \!+\! {\lambda _2}L_{{\text {Style}}}^{{\text {Lumi}}} \!+\! {\lambda _3}L_{{\text {Valid}}}^{{\text {Lumi}}} \!+\! {\lambda _4}L_{{\text {Hole}}}^{{\text {Lumi}}}, \end{aligned}$$

where ${\lambda } _{1}$, ${\lambda } _{2}$, ${\lambda }_{3}$ and ${\lambda }_{4}$ are the weighting parameters, ${L_{\mathrm{{Valid}}}^{\mathrm{{Lumi}}}}$ is the ${\ell _1}$ loss on the valid areas, and ${L_{\mathrm{{Hole}}}^{\mathrm{{Lumi}}}}$ is the ${\ell _1}$ loss on the damaged areas. Analogously, the total loss for the sketch reasoning module is:

20 $$\begin{aligned} {L_{{\text {Sketch}}}} \!=\! {\lambda _5}L_{{\text {Percept}}}^{{\text {Sketch}}} \!+\! {\lambda _6}L_{{\text {Style}}}^{{\text {Sketch}}} \!+\! {\lambda _7}L_{{\text {Valid}}}^{{\text {Sketch}}} \!+\! {\lambda _8}L_{{\text {Hole}}}^{{\text {Sketch}}}, \end{aligned}$$

where ${\lambda } _{5}$, ${\lambda } _{6}$, ${\lambda }_{7}$ and ${\lambda }_{8}$ are the weighting parameters, ${L_{\mathrm{{Valid}}}^{\mathrm{{Sketch}}}}$ is the ${\ell _1}$ loss on the valid areas, and ${L_{\mathrm{{Hole}}}^{\mathrm{{Sketch}}}}$ is the ${\ell _1}$ loss on the damaged areas.

Lastly, the total loss for the color fusion module is defined as:

21 $$\begin{aligned} {L_{\mathrm{{Color}}}} \!=\! {\tau _1}L_{\mathrm{{Percept}}}^{\mathrm{{Color}}} \!+\! {\tau _2}L_{\mathrm{{Style}}}^{\mathrm{{Color}}} \!+\! {\tau _3}L_{\mathrm{{Valid}}}^{\mathrm{{Color}}} \!+\! {\tau _4}L_{\mathrm{{Hole}}}^{\mathrm{{Color}}}, \end{aligned}$$

where ${\tau _1},{\tau _2},{\tau _3}$ and ${\tau _4}$ are the weighting parameters. This loss function construction aims to restore missing regions by learning color information from valid areas in damaged images.

## Experiments

### Datasets

**Fig. 4:**
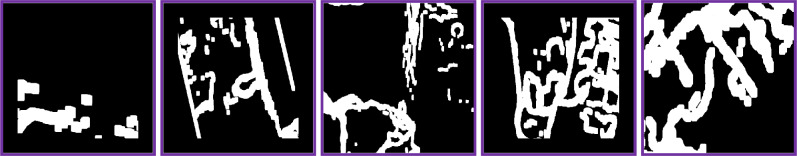
Examples of NVIDIA’s irregular mask dataset.

Mural image restoration requires a large amount of high-quality training data, but only a few mural images are available in practice. Some deep learning methods may perform poorly in image completion due to small mural data. Therefore, we select a large number of natural images as auxiliary data to train the network models because natural images are somewhat similar to mural images. We collected two distinct image datasets to train and evaluate the proposed PRN model. One was the widely used Places2^36^ dataset, comprising 1.8 million training images from 365 scenario categories, with 50 validation images and 900 testing images per category. The resolution of each image in the Places2 dataset is 256$\times$256. The other dataset was our self-made dataset ‘Murals2’ divided into 300 images for training, 100 images for validation, and 100 images for testing. Each image of the Murals2 dataset was cropped to a resolution of 256$\times$256. To produce realistic missing areas, we used NVIDIA’s irregular mask dataset^13^ to simulate various missing shapes. This mask dataset contains 55116 masks for training, 6000 masks for validation, and 6000 masks for testing. According to the proportion of missing areas in a mask, the testing masks were further categorized into six subsets of the same size, located in the respective intervals (0.01,0.1], (0.1,0.2], (0.2,0.3], (0.3,0.4], (0.4,0.5], and (0.5,0.6]. During the training and validation phases, for each image (regarded as the ground-truth image) selected from the respective image dataset, we randomly chose a mask from the mask dataset. We then applied an element-wise product between the image and the mask to simulate a damaged image. Figure 4 exemplifies four samples from NVIDIA’s irregular mask dataset. As indicated in Fig. 4, white regions in the masks represent missing areas of the images, whereas black regions represent the valid portions. Given the scarcity of mural images, we adopted transfer learning to train all network models for fair comparisons. Specifically, we first trained each model using cross-validation on the Places2 dataset and then fine-tuned it on the Murals2 dataset. Both the initially trained and fine-tuned models were evaluated on the testing images to validate their efficacy.

### Experimental settings

We introduced hyper-parameters in our model, specifically the weighting parameters in Eqs. (6), (19), (20) and (21). In Eq. (6), we employed $\lambda \in \left[ {0,1}\right]$ to strike a balance between the effects of consecutive iterations. During the training phase, we incrementally adjusted $\lambda$ from 0 to 1 in increments of 0.1, while keeping all other hyper-parameters fixed. Our observations indicated that the optimal performance and the fastest convergence were achieved when $\lambda =0.5$. In Eq. (19), we adjusted the values of ${\lambda } _{1}$, ${\lambda }_{2}$, ${\lambda }_{3}$ and ${\lambda }_{4}$ to find the ideal balance among the four components of the total loss function. The model exhibited convergence when all weighted terms aligned closely. Through rigorous testing and adjustment, we settled on the following values: ${\lambda } _{1}=0.05$, ${\lambda }_{2}=120$, ${\lambda }_{3}=1$ and ${\lambda }_{4}=6$. Likewise, for Eqs. (20) and (21), we determined the optimal hyper-parameters to be ${\lambda } _{5}=0.05$, ${\lambda }_{6}=120$, ${\lambda }_{7}=1$, ${\lambda }_{8}=6$, ${\tau } _{1}=0.05$, ${\tau }_{2}=100$, ${\tau }_{3}=1$ and ${\tau }_{4}=5$.

The parameters of the bilateral filter were configured as follows: a neighborhood diameter of $d=9$, a color space filter sigma of ${\sigma _{\mathrm{{Color}}}} = 60$, and a coordinate space filter sigma of ${\sigma _{\mathrm{{Coord}}}} = 9$. We adopted the adaptive moment estimation (Adam) optimizer^37^ for training our PRN model with a batch size of 6 by transfer learning. The hyper-parameters of Adam were set as $\varepsilon = {10^{-8}}$, ${\beta _1} = 0.9$ and ${\beta _2} = 0.999$. For transfer learning, the training process consisted of two stages. Initially, the model was trained on the Places2 dataset with successive learning rates of $2 \times {10^{-4}}$ and $5 \times {10^{-5}}$. Subsequently, the model underwent fine-tuning on the Murals2 dataset using learning rates of $1\times {10^{-4}}$ and $2\times {10^{-5}}$. Our PRN was implemented with PyTorch, and its three modules were trained on separate NVIDIA TITAN Xp GPUs. The luminance, sketch reasoning, and color fusion modules were trained for 10, 4, and 2 days on the Places2 dataset, followed by fine-tuning for 2, 1, and 1 days on the Murals2 dataset, respectively. The final, well-trained model was selected through cross-validation for testing.

### Ablation study

To validate the effectiveness of each component within our PRN, we rearranged its three modules into three distinct models for our ablation study. The first model variant (named PRN-C) eliminates the color fusion module and relies solely on the luminance and sketch reasoning modules to estimate the complete true-color images. The second variant (named PRN-A) retains all other components but replaces the second codec with an averaging operation across all cyclic double-codec structures. The last variant (named PRN-D) is constructed by removing the differential reinforcement block from the color fusion module. We conducted a comparative analysis of our PRN against these three variants using testing images from both the Places2 and Murals2 datasets.

**Fig. 5:**
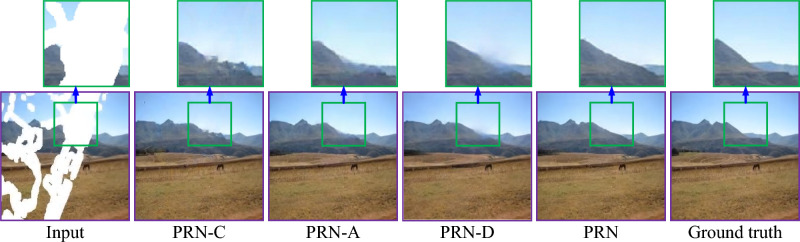
Visual comparison of repaired images obtained by our PRN and its variants for a testing image randomly selected from the Places2 dataset.

**Fig. 6:**
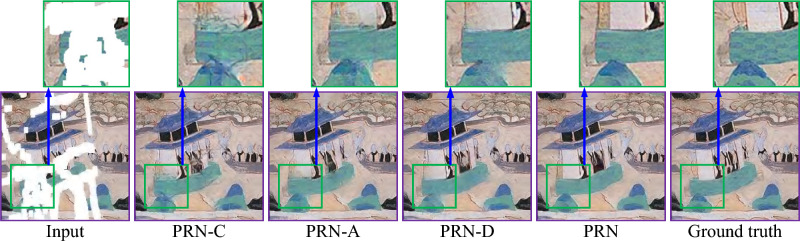
Visual comparison of repaired images obtained by our PRN and its variants for a testing image randomly selected from the Murals2 dataset.

Figures 5 and 6 show a visual comparison between the repaired images obtained by our PRN and its variants, using two testing images randomly selected from the respective Places2 and Murals2 datasets. PRN-C produces significant distortion in the repaired images. Both PRN-A and PRN-D cause abundant artifacts and blurred regions within the repaired images. In contrast, our PRN recovers more accurate colors and clearer image details than its three variants.

Table 1 shows the peak signal-to-noise ratio (PSNR, measured in dB), structural similarity index measure (SSIM), and mean ${\ell _1}$ error values for the repaired images. These images were obtained by our PRN and its three variants on testing images corrupted with various mask ratios. As Table 1 demonstrates, our PRN consistently outperforms its variants across all three metrics: PSNR, SSIM, and mean ${\ell _1}$ norm. This strongly suggests that the color fusion module, double-codec components, and the differential reinforcement block are all crucial and advantageous elements of our PRN.

### Baseline methods

To evaluate the performance of the proposed model, we selected milestone or popular methods as baseline methods. They include CR-Fill^38^, TFill^39^, LGNet^40^, AOT-GAN^23^, SCAT^41^, CMT^42^, M2S^43^ and HINT^44^. For a fair comparison, we utilized the experimental results either reported in the papers or reproduced from the source codes released by the authors. All baseline methods were fine-tuned based on the publicly available pre-trained weights. Both subjective and objective evaluations were conducted to compare the proposed model and baseline methods.

### Method evaluation

We compared the proposed PRN with various state-of-the-art methods, including CR-Fill^38^, TFill^39^, LGNet^40^, AOT-GAN^23^, SCAT^41^, CMT^42^, M2S^43^ and HINT^44^. This comparison was conducted both qualitatively and quantitatively on the widely used Places2 and our customized Murals2 datasets. For the evaluation, we used the source codes provided by the authors of these baseline methods. To ensure a fair comparison, all methods underwent training and testing on the same image datasets. The hyperparameter settings for the comparison methods followed those specified by their respective authors. If a pre-trained model was available, we performed transfer learning based on that model, fine-tuning it on the Murals2 dataset.

**Table 1:** Quantitative evaluation values of repaired images obtained by our PRN and its variants for testing images from both the Places2 and Murals2 datasets.

Mask ratio		$\left( {0.1,0.2} \right]$		$\left( {0.3,0.4} \right]$		$\left( {0.5,0.6} \right]$	
Dataset		Places2	Murals2	Places2	Murals2	Places2	Murals2
PSNR $\uparrow$	PRN-C	28.32	27.08	22.99	22.18	20.37	19.80
	PRN-A	28.62	27.40	23.47	22.61	20.76	20.15
	PRN-D	28.70	27.44	23.42	22.68	20.97	20.24
	Our PRN	**28.77**	**27.56**	**23.58**	**22.79**	**21.06**	**20.35**
SSIM $\uparrow$	PRN-C	0.9392	0.9206	0.8208	0.7950	0.5883	0.5671
	PRN-A	0.9432	0.9235	0.8256	0.8028	0.6110	0.5815
	PRN-D	0.9439	0.9238	0.8269	0.8046	0.6126	0.5877
	Our PRN	**0.9451**	**0.9258**	**0.8293**	**0.8061**	**0.6156**	**0.5905**
Mean ${\ell _1}$ $\downarrow$	PRN-C	1.12	1.54	2.73	3.15	6.11	7.16
	PRN-A	1.05	1.46	2.57	3.03	5.91	6.92
	PRN-D	1.03	1.41	2.50	2.91	5.80	6.87
	Our PRN	**1.01**	**1.36**	**2.42**	**2.86**	**5.71**	**6.78**

$\uparrow$ Higher is better. $\downarrow$ Lower is better. The best results are highlighted in bold.

**Fig. 7:**
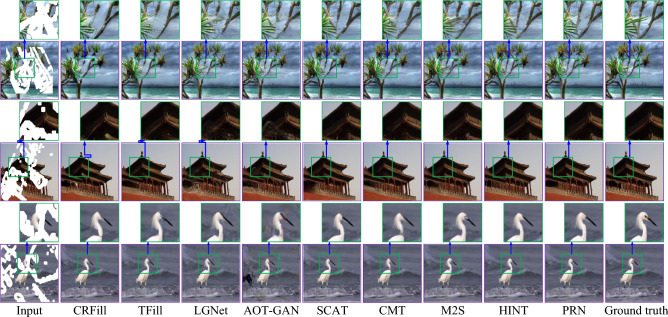
Visual comparison of repaired images obtained by CR-Fill^38^, TFill^39^, LGNet^40^, AOT-GAN^23^, SCAT^41^, CMT^42^, M2S^43^, HINT^44^ and our PRN for three testing images randomly selected from the Places2 dataset.

**Fig. 8:**
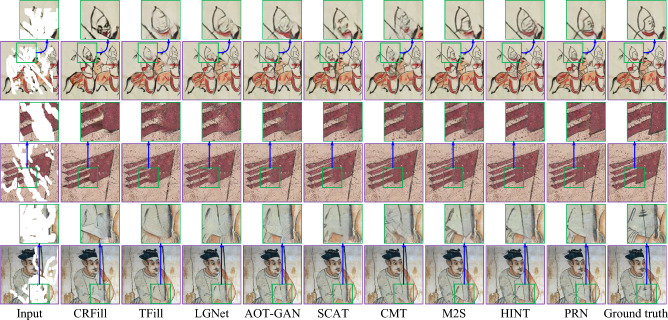
Visual comparison of repaired images obtained by CR-Fill^38^, TFill^39^, LGNet^40^, AOT-GAN^23^, SCAT^41^, CMT^42^, M2S^43^, HINT^44^ and our PRN for three testing images randomly selected from the Murals2 dataset.

Figures 7 and 8 show a visual comparison of the repaired images produced by baseline methods^23,38–44^ and the proposed PRN for the simulated testing images. These images were randomly chosen from the Places2 and Murals2 datasets, respectively. As depicted in the figures, CR-Fill^38^ tends to introduce excessive smoothness, resulting in a loss of details. TFill^39^, LGNet^40^ and AOT-GAN^23^ exhibit some local blurring and structural inaccuracies, while SCAT^41^ and HINT^44^ also produce blurry details. Although M2S^42^ produces better repair results than other comparison methods, the sketches in its repaired images are still blurred. For example, the lines of tree trunks are blurred, and the lines on the lower left corner of the flag are missing. In contrast, our PRN consistently demonstrates superior repair results, delivering clear structures and realistic colors, thereby enhancing visual comfort compared to baseline methods.

**Table 2:** Quantitative evaluation values of repaired images obtained by our PRN and baseline methods for testing images from the Places2 and Murals2 datasets.

Mask Ratio		$\left( {0.1,0.2} \right]$		$\left( {0.3,0.4} \right]$		$\left( {0.5,0.6} \right]$	
Dataset		Places2	Murals2	Places2	Murals2	Places2	Murals2
PSNR $\uparrow$	CR-Fill [2021]	28.39	27.16	23.08	22.32	20.56	19.97
	TFill [2022]	28.57	27.25	23.33	22.57	20.77	20.10
	LGNet [2022]	28.28	27.03	22.94	22.21	20.42	19.88
	AOT-GAN [2023]	28.21	26.91	22.82	22.14	20.30	19.79
	SCAT [2023]	28.62	27.14	23.19	22.47	20.65	19.64
	CMT [2023]	28.75	27.35	23.25	22.53	20.73	19.72
	M2S [2024]	28.91	27.44	23.49	22.61	20.94	19.95
	HINT [2024]	**29.01**	27.52	23.53	22.71	20.89	19.96
	PRN	28.77	**27.56**	**23.58**	**22.79**	**21.06**	**20.35**
SSIM $\uparrow$	CR-Fill [2021]	0.9419	0.9215	0.8234	0.7984	0.6009	0.5782
	TFill [2022]	0.9430	0.9231	0.8257	0.8021	0.6056	0.5668
	LGNet [2022]	0.9411	0.9211	0.8219	0.7963	0.5944	0.5596
	AOT-GAN [2023]	0.9395	0.9198	0.8191	0.7940	0.5831	0.5502
	SCAT [2023]	0.9353	0.9115	0.8199	0.8045	0.6054	0.5849
	CMT [2023]	0.9375	0.9263	0.8247	0.7936	0.6073	0.5732
	M2S [2024]	0.9531	**0.9379**	0.8288	0.8042	0.6145	0.5875
	HINT [2024]	**0.9535**	0.9342	0.8276	0.7859	0.6038	0.5810
	PRN	0.9451	0.9258	**0.8293**	**0.8061**	**0.6156**	**0.5905**
Mean ${\ell _1}$ $\downarrow$	CR-Fill [2021]	1.08	1.45	2.60	3.04	5.94	7.03
	TFill [2022]	1.04	1.41	2.52	2.98	5.91	6.90
	LGNet [2022]	1.11	1.49	2.65	3.10	6.11	7.06
	AOT-GAN [2023]	1.13	1.52	2.71	3.14	6.19	7.17
	SCAT [2023]	1.06	1.47	2.54	3.11	6.02	7.11
	CMT [2023]	1.10	1.31	2.51	2.97	5.94	6.54
	M2S [2024]	1.05	1.24	2.36	2.91	5.84	6.39
	HINT [2024]	1.07	**1.23**	2.68	3.01	5.80	6.78
	PRN	**1.01**	1.36	**2.42**	**2.86**	**5.71**	**6.01**
LPIPS $\downarrow$	CR-Fill [2021]	0.0896	0.0811	0.1821	0.1796	0.2907	0.3214
	TFill [2022]	0.0897	0.0826	0.1815	0.1874	0.2913	0.3319
	LGNet [2022]	0.0872	0.0839	0.1784	0.1857	0.2965	0.3461
	AOT-GAN [2023]	0.0843	0.0813	0.1771	0.1831	0.2943	0.3076
	SCAT [2023]	0.0824	0.0828	0.1779	0.1822	0.2944	0.3151
	CMT [2023]	0.0819	0.0783	0.1754	0.1804	0.2931	0.3102
	M2S [2024]	0.0796	**0.0765**	0.1637	0.1722	0.2849	0.3075
	HINT [2024]	0.0794	0.0794	0.1644	0.1737	**0.2804**	0.3084
	PRN	**0.0782**	0.0772	**0.1602**	**0.1709**	0.2816	**0.3064**

$\uparrow$ Higher is better. $\downarrow$ Lower is better. The best results are highlighted in bold.

We also compared our PRN with baseline methods quantitatively. For testing images from the Places2 and Murals2 datasets, Table 2 presents PSNR (dB), SSIM, mean ${\ell _1}$ error, and learned perceptual image patch similarity (LPIPS) values for the repaired images obtained by our PRN and baseline methods. The mask ratio denotes the proportion of missing areas relative to the entire image. As shown in Table 2, for the small mask ratio (0.1, 0.2], our PRN achieves lower PSNR/SSIM but lower mean ${\ell _1}$ and LPIPS than M2S^43^ and HINT^44^ on the Places2 dataset. In contrast, our PRN achieves higher PSNR/mean ${\ell _1}$ but lower SSIM than M2S^43^ and HINT^44^. Additionally, our PRN has lower LPIPS than HINT^44^, but higher LPIPS than M2S^43^ on the Murals2 dataset. Furthermore, for the large mask ratio (0.5, 0.6], our PRN has higher LPIPS than HINT^44^ on the Places2 dataset. Therefore, our PRN generally outperforms baseline methods across all four metrics.

**Fig. 9:**
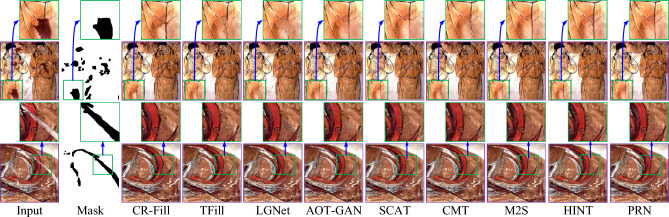
Visual comparison of repaired images obtained by CR-Fill^38^, TFill^39^, LGNet^40^, AOT-GAN^23^, SCAT^41^, CMT^42^, M2S^43^ and HINT^44^ and our PRN for real damaged mural images.

In addition to evaluating simulated damaged images, we also assessed the performance of our PRN on real damaged mural images. Figure 9 offers a visual comparison between the repaired images obtained by baseline methods and our PRN for these real damaged murals. It is evident from Fig. 9 that our PRN excels in restoring both small and large missing areas, demonstrating remarkable robustness and consistency across diverse scenes.

### Computational complexity

In addition to qualitative and quantitative performance evaluations, we conducted a comprehensive analysis of the computational complexity of our PRN compared to baseline methods. Our PRN comprises luminance, sketch, and color fusion modules. Both luminance and sketch reasoning modules share a cyclic double-codec structure. Each codec has 18 convolutional layers, with the former codec incorporating an attention layer at its bottleneck and 4 partial convolutional layers at the encoder’s front end. The former codec of each cyclic double-codec structure performs 6 iterations, generating 6 pairs of initial luminance and sketch maps. These are then fused by the latter codec to produce a final pair of luminance and sketch maps. The color fusion module includes 10 gated convolutional layers, 5 convolutional layers, 6 spatial attention layers, and 3 channel attention layers. Therefore, the total computational complexity of PRN, measured in floating point operations (FLOPs), is approximated by the following equation:

22 $$\begin{aligned} \textrm{Time} \sim o\left( {9.7HW \times {{10}^6}} \right) , \end{aligned}$$

where *H* and *W* are the height and width of the input image, respectively.

To evaluate the practical performance of our PRN, We conducted tests on an NVIDIA RTX 2060 GPU (12GB) using Python 3.9 under the Windows 11 operating system, powered by an Intel Core i5-12400 chip (16GB RAM, 512GB SSD). Table 3 compares the computation time of each module within our PRN for two randomly selected testing images of respective sizes $256\times 256\times 3$ and $512\times 512\times 3$.

**Table 3:** Computation time of each module in our PRN (unit: seconds).

Image size	Luminance reasoning	Sketch reasoning	Color fusion	Total time
$256\times 256\times 3$	0.88	0.88	0.73	2.49
$512\times 512\times 3$	1.34	1.34	0.81	3.49

**Table 4:** Comparison of computation time between our PRN and baseline methods (unit: seconds).

Image Size	CR-Fill	TFill	LGNet	AOT-GAN	SCAT	CMT	M2S	HINT	PRN
$256\times 256\times 3$	1.54	2.95	0.07	0.84	0.29	0.96	1.36	0.47	2.49
$512\times 512\times 3$	1.62	4.61	0.36	1.17	0.31	1.73	2.48	0.62	3.49

Furthermore, Table 4 presents a comparison of computation times between our PRN and various baseline methods. While our unoptimized PRN takes longer than CR-Fill^38^, LGNet^40^, AOT-GAN^23^, SCAT^41^, CMT^42^, M2S^43^ and HINT^44^, it still outperforms TFill^39^ in terms of computation time. Similar results were observed for other testing images. These findings demonstrate the computational efficiency of our PRN relative to some baseline methods, highlighting its potential for practical applications despite its complexity.

## Discussion

We decomposed the complex image completion problem into three progressive subtasks rather than treating it as a whole optimization task. This can make it easier to train the model, execute the task, and improve the performance of image completion. Figures 5 and 6 and Tables 1 and 2 demonstrate the rationality and effectiveness of our PRN model. From Figs. 7, 8 and 9, our PRN produces visually pleasing repaired images and demonstrates better repair results than CR-Fill^38^, TFill^39^, LGNet^40^, AOT-GAN^23^, SCAT^41^ and CMT^42^. However, our PRN may generate more blurry lines and artifacts in some cases than M2S^43^ and HINT^44^. This could be due to model forecast errors in missing data estimation. Moreover, our PRN often achieves superior repair results compared to baseline methods, albeit with increased computational complexity, as illustrated in Table 4. Through the experiments, we discovered that the consistency between the estimated luminance and sketch maps is crucial for achieving high-quality image completion. To ensure this consistency, we utilized a unified network architecture and datasets in our methodology. Considering the trade-off between computational complexity and performance, our PRN shows great potential for applications in the field of digital art preservation and restoration.

## Conclusion

In this paper, we present a novel mural image completion model based on the progressive reasoning network. This model incorporates luminance and sketch reasoning modules, both constructed on the same cyclic double-codec frameworks, aimed at estimating a matching pair of luminance and sketch maps. Additionally, we designed a color fusion module that utilizes differential reinforcement and residual attention blocks to reconstruct the complete true-color image. By employing transfer learning, the model is trained on both publicly available and customized datasets. Experimental results reveal that our proposed model not only produces realistic repaired images but also outperforms the state-of-the-art methods, both qualitatively and quantitatively. To further enhance image completion performance, we intend to conduct comprehensive research on repairing issues such as pigment shedding and other diseases affecting digitized images of ancient murals.

## Data Availability

The Places2 dataset is available from: http://places2.csail.mit.edu. The Murals2 dataset used and analyzed during the current study is available from the corresponding author upon reasonable request.
